# Teclistamab Monotherapy in Relapsed or Refractory Multiple Myeloma: A Real-World Focused Systematic Review and Meta-Analysis of Efficacy and Safety

**DOI:** 10.3390/hematolrep18040054

**Published:** 2026-08-03

**Authors:** Jerry Qi, Daniel Park, Pranati Shah, Mojtaba Akhtari

**Affiliations:** 1 Department of Medicine, Loma Linda University Medical Center, 11234 Anderson Street, Loma Linda, CA 92354, USA; 2 Department of Medicine, University of California, San Francisco, Fresno Campus, 155 N. Fresno Street, Fresno, CA 93701, USA; 3 Division of Transplant, Cellular Therapy, and Hematological Malignancies, Department of Medicine, Loma Linda University Medical Center, 11234 Anderson Street, Loma Linda, CA 92354, USA

**Keywords:** multiple myeloma, relapsed/refractory multiple myeloma, teclistamab, bispecific antibody, BCMA, meta-analysis

## Abstract

**Background/Objectives:** Teclistamab is a B-cell maturation antigen (BCMA)-directed bispecific antibody approved for relapsed or refractory multiple myeloma (RRMM). Although prior analyses have summarized teclistamab outcomes, some have included combination regimens or broader comparative studies. Given the expanding real-world experience with teclistamab, we conducted a systematic review and meta-analysis focused on teclistamab monotherapy in RRMM. **Methods:** We performed a systematic review and meta-analysis of clinical trials and retrospective real-world studies evaluating teclistamab monotherapy in RRMM. The primary endpoint was overall response rate (ORR). Secondary endpoints included complete response (CR), overall mortality rate (OMR), mortality due to multiple myeloma progression, teclistamab-attributed mortality, adverse event-related mortality, grade ≥3 cytokine release syndrome (CRS), grade ≥3 immune effector cell-associated neurotoxicity syndrome (ICANS), and other grade ≥3 adverse events. Pooled proportions were estimated using random-effects models. **Results:** Twelve studies including 761 patients were analyzed, comprising two clinical trials and ten real-world retrospective cohorts. The pooled ORR was 60.9%, and the pooled CR rate was 25.2%. The pooled OMR was 26.9%, with mortality due to myeloma progression of 23.9%, adverse event-related mortality of 8.1%, and teclistamab-attributed mortality of 3.3%. Severe (grade ≥3) CRS and ICANS were uncommon, with grade ≥3 events occurring in 1.7% and 3.1%, respectively. Grade ≥3 infections occurred in 28.3%. Common grade ≥3 hematologic adverse events included lymphopenia, neutropenia, anemia, and thrombocytopenia. **Conclusions:** Teclistamab monotherapy demonstrates meaningful clinical activity and a manageable immune toxicity profile in heavily pretreated RRMM. However, severe infections and hematologic toxicities remain important clinical considerations, supporting the need for close monitoring, infection prevention, and supportive care during therapy.

## 1. Introduction

Multiple myeloma (MM) is a hematologic malignancy with an increasing global incidence over the past three decades and accounts for nearly 2% of all new cancer diagnoses and cancer-related mortality in the United States [1,2]. It is characterized by the clonal proliferation of malignant plasma cells within the bone marrow, leading to end-organ damage including anemia, lytic bone lesions, renal dysfunction, hypercalcemia, and cytopenias [3]. Standard frontline therapy for newly diagnosed MM typically includes combinations of immunomodulatory agents, proteasome inhibitors, and corticosteroids, with the addition of anti-CD38 monoclonal antibodies further improving clinical outcomes [4,5]. Despite these advances, MM remains incurable, and most patients ultimately experience disease relapse and progression.

Relapsed or refractory multiple myeloma (RRMM) represents a major therapeutic challenge, particularly in heavily pretreated patients who have developed resistance to multiple drug classes. These patients typically have limited treatment options and poor outcomes. In this setting, B-cell maturation antigen (BCMA) has emerged as a promising therapeutic target due to its high and selective expression on malignant plasma cells and its role in disease progression [6]. Several BCMA-directed therapies have since been developed, including antibody–drug conjugates, chimeric antigen receptor T-cell (CAR-T) therapies, and bispecific antibodies.

Teclistamab is a bispecific antibody targeting both CD3 on T cells and BCMA on myeloma cells, resulting in T-cell-mediated cytotoxicity of BCMA-expressing myeloma cells [7,8,9]. In the pivotal MajesTEC-1 clinical trial, teclistamab demonstrated meaningful response rates in heavily pretreated RRMM patients with a manageable safety profile [9]. However, clinical trial populations may not fully reflect patients treated in routine clinical practice, where individuals are often older, have a greater comorbidity burden, or have clinical features that would have limited trial eligibility.

Recent meta-analyses have summarized the efficacy and safety of teclistamab in RRMM, including broader analyses that incorporated combination regimens [10,11]. However, as teclistamab monotherapy remains an important treatment approach, an updated product-specific synthesis focused specifically on teclistamab monotherapy may provide clinically useful estimates of efficacy and safety. Integrating both clinical trial and real-world data may also help contextualize outcomes across diverse patient populations and treatment settings. Therefore, we conducted a systematic review and meta-analysis evaluating the efficacy and safety of teclistamab monotherapy in patients with RRMM across clinical trials and real-world studies.

## 2. Methods

### 2.1. Search Strategy

**Figure 1:**
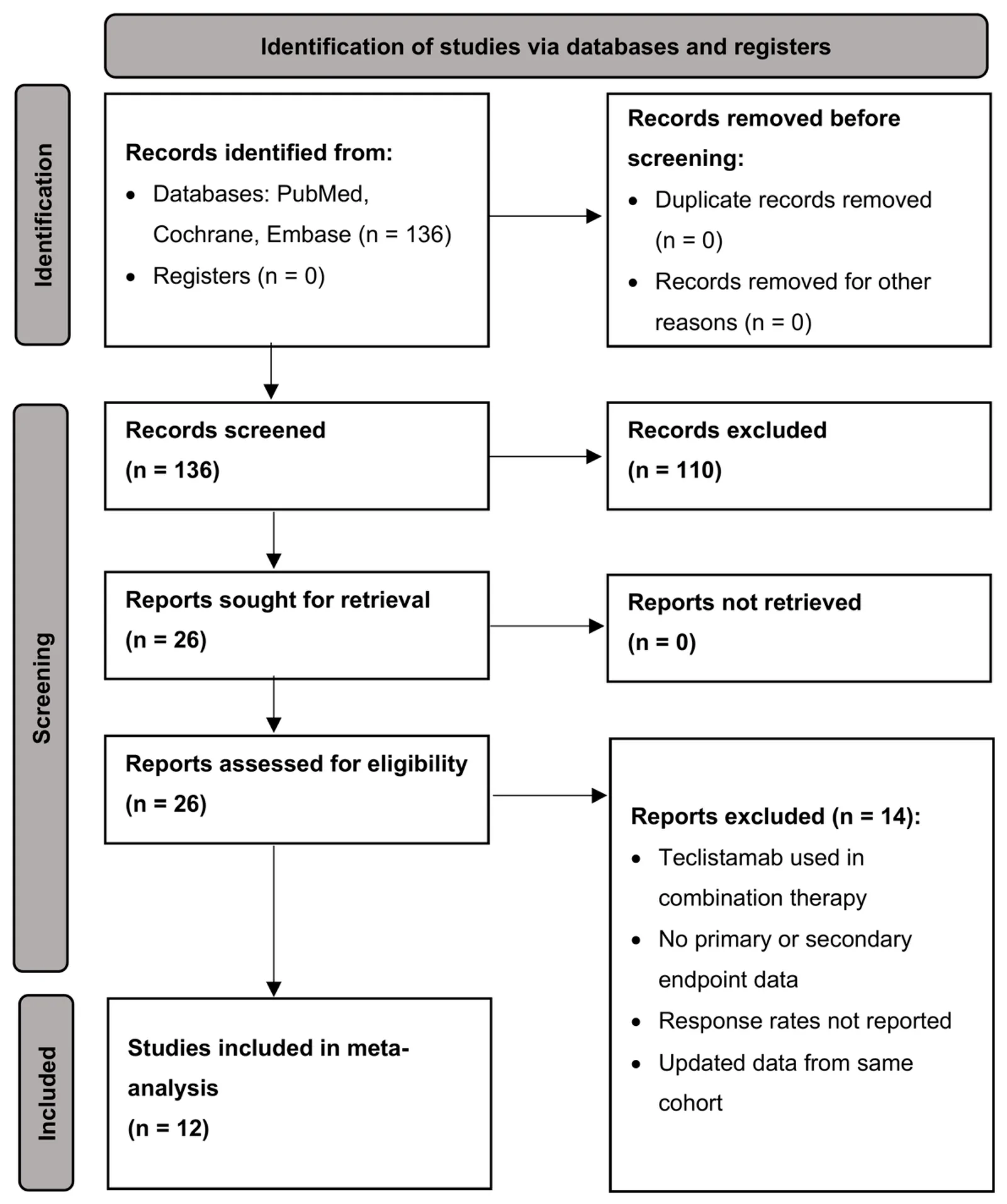
PRISMA flow diagram of study selection.

We conducted a literature search using the PICOS (Population, Interventions, Comparators, Outcomes, and Study Design) model. Preferred Reporting Items for Systematic Reviews and Meta-Analyses (PRISMA) 2020 guidelines were followed [12]. The PRISMA 2020 checklist is provided in Supplementary Table S3. This review was not prospectively registered. A formal review protocol was not prepared. PubMed, Embase, and the Cochrane Library were used to identify studies reporting teclistamab monotherapy in RRMM. The literature search was last updated on 17 October 2024. Eligible study designs included single-center and multi-center clinical trials and retrospective studies. The search terms included “teclistamab”, “monotherapy”, “multiple myeloma”, “relapsed refractory multiple myeloma”, and “teclistamab adverse events”. Inclusion criteria were clinical trials and retrospective studies reporting teclistamab monotherapy in patients with RRMM. Exclusion criteria were studies and trials utilizing teclistamab in other disease settings, in previously untreated MM, or in combination therapies. Studies without primary or secondary endpoints, without response rate reporting, or reporting on updated results from a previously reported cohort were also excluded. Case reports, literature reviews, and letters to the editor were excluded. Study selection was performed independently by two authors, and any discrepancies were resolved by a third reviewer. Data extraction was completed independently by two authors and reviewed by a third author. Figure 1 summarizes the study selection process for this meta-analysis.

### 2.2. Risk of Bias Assessment

Study-level risk of bias for retrospective real-world studies was assessed using a modified Newcastle–Ottawa Scale (NOS) adapted for single-arm cohort designs [13]. The assessment included domains related to cohort selection, exposure ascertainment, baseline outcome status, comparability, outcome assessment, follow-up duration, and follow-up completeness. Because the real-world studies included in this review evaluated teclistamab as single-arm cohorts, the non-exposed cohort domain was considered not applicable and was not awarded a star. Comparability was credited when studies analyzed or adjusted for clinically relevant prognostic factors, such as age, prior BCMA-directed therapy, extramedullary disease, cytogenetic risk, or other high-risk disease features. Studies were scored out of a maximum of 9 points and were categorized as low risk of bias for scores of 7–9, moderate risk for scores of 4–6, and high risk for scores of 0–3. The modified NOS was applied only to retrospective real-world studies; prospective clinical trials were not evaluated using this tool.

### 2.3. Certainty of Evidence Assessment

Certainty of evidence for key pooled outcomes was assessed using the GRADE framework [14]. Because the included studies were predominantly single-arm studies of teclistamab monotherapy, certainty was interpreted as confidence in pooled absolute estimates rather than comparative treatment effects. Certainty was assessed separately for each outcome across the domains of risk of bias, inconsistency, indirectness, imprecision, and publication bias. Risk-of-bias judgments within the GRADE assessment were informed by the modified Newcastle–Ottawa Scale assessment.

### 2.4. Primary and Secondary Outcomes

The primary endpoint of the study was overall response rate (ORR). Secondary endpoints were complete response (CR), overall mortality rate (OMR; all-cause mortality), mortality rate due to multiple myeloma progression, mortality rate due to teclistamab, mortality rate due to adverse events, and rate of reported grade ≥3 adverse events (AEs). Rates of grade ≥3 cytokine release syndrome (CRS) and immune effector cell-associated neurotoxicity syndrome (ICANS) were also analyzed.

### 2.5. Statistical Methods

Meta-analyses of proportions were performed to estimate pooled proportions of ORR, CR, OMR, mortality outcomes, and adverse event profiles across included studies. Outcomes were pooled when reported by included studies; outcomes not reported in a given study were excluded from that endpoint-specific synthesis. Because substantial heterogeneity between studies was expected, all analyses were performed using a random-effects model. No variance-stabilizing transformation, including Freeman–Tukey double arcsine transformation, was applied when pooling proportions. Individual 95% confidence intervals were calculated using Clopper–Pearson intervals for binomial proportions. Heterogeneity was evaluated using the I^2^ statistic, which summarizes the percentage of variance explained by heterogeneity compared to random chance. Any outcome with an I^2^ ≥ 60% represented high heterogeneity. Meta-analyses were performed using the meta package in R. Formal statistical assessment of small-study effects or publication bias and sensitivity analyses were not performed.

## 3. Results

Twelve clinical studies, including two clinical trials and ten real-world retrospective studies, were selected for this meta-analysis, with a total of 761 patients. Table 1 describes the baseline characteristics of the included patients.

The modified Newcastle–Ottawa Scale was applied to the ten retrospective real-world studies (Supplementary Table S1). Scores ranged from 6 to 8 out of 9, corresponding to low-to-moderate risk of bias. Five studies were rated as having low risk of bias, with scores ranging from 7 to 8 out of 9, and five studies were rated as having moderate risk of bias, each scoring 6 out of 9. No retrospective study was categorized as high risk. Across studies, common strengths generally included clearly defined teclistamab exposure, reporting of baseline disease and treatment characteristics, use of established response and toxicity criteria when specified, and follow-up that was adequate for early efficacy and safety endpoints. Key limitations included the lack of non-exposed comparator cohorts, limited adjustment for confounding, short follow-up in several studies, and abstract-only reporting for some cohorts without full methodological details.

### 3.1. Evaluation of Efficacy

**Figure 2:**
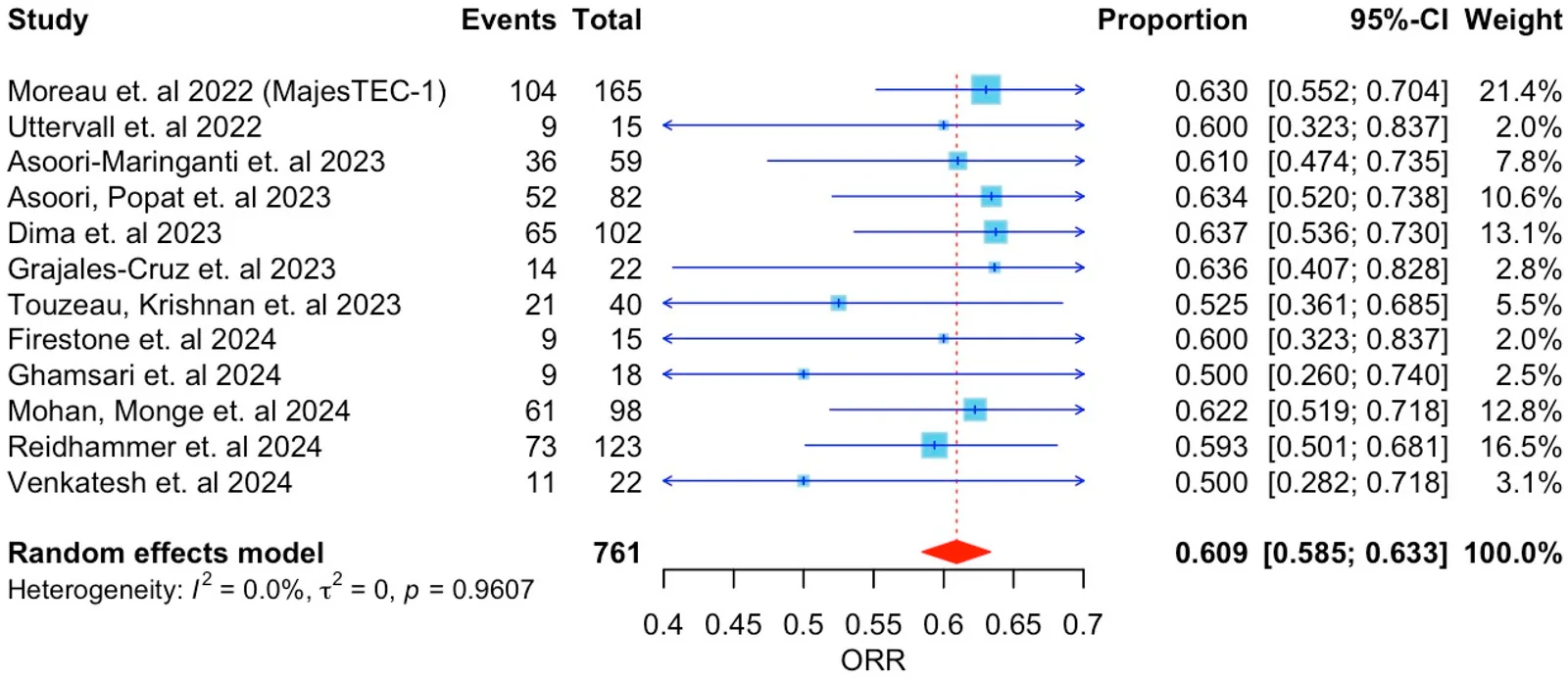
Forest plot of pooled ORR: 60.9% (95% CI 58.5–63.3%, I^2^ = 0.0%, *p* = 0.96). Data were derived from contributing studies [9,15,16,17,18,19,20,21,22,23,24,25].

**Figure 3:**
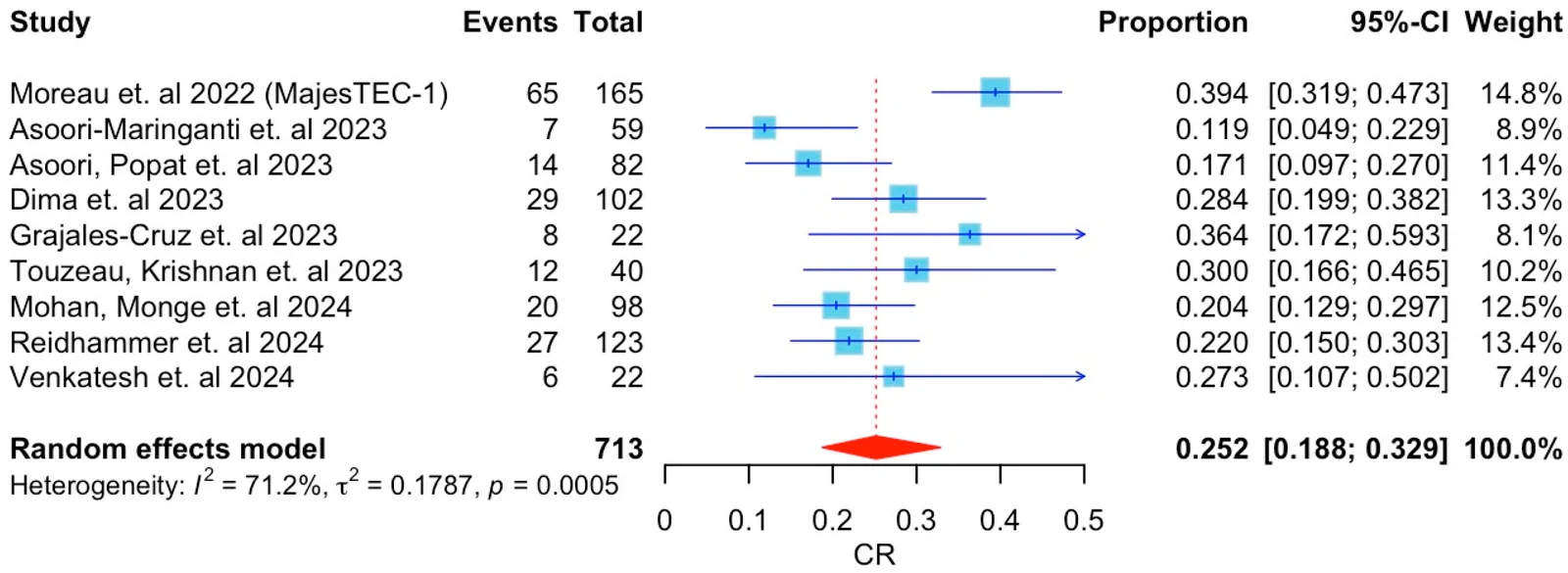
Forest plot of pooled CR: 25.2% (95% CI 18.8–32.9%, I^2^ = 71.2%, *p* = 0.0005). Data were derived from contributing studies [9,16,17,18,19,20,23,24,25].

The ORR of teclistamab monotherapy in the treatment of RRMM was assessed in all 12 studies. The pooled ORR was 60.9% (95% confidence interval [CI] 58.5–63.3%, I^2^ = 0.0%, *p* = 0.96) (Figure 2). CR was assessed in 9 studies. The pooled CR rate was 25.2% (95% CI 18.8–32.9%, I^2^ = 71.2%, *p* = 0.0005) (Figure 3).

**Figure 4:**
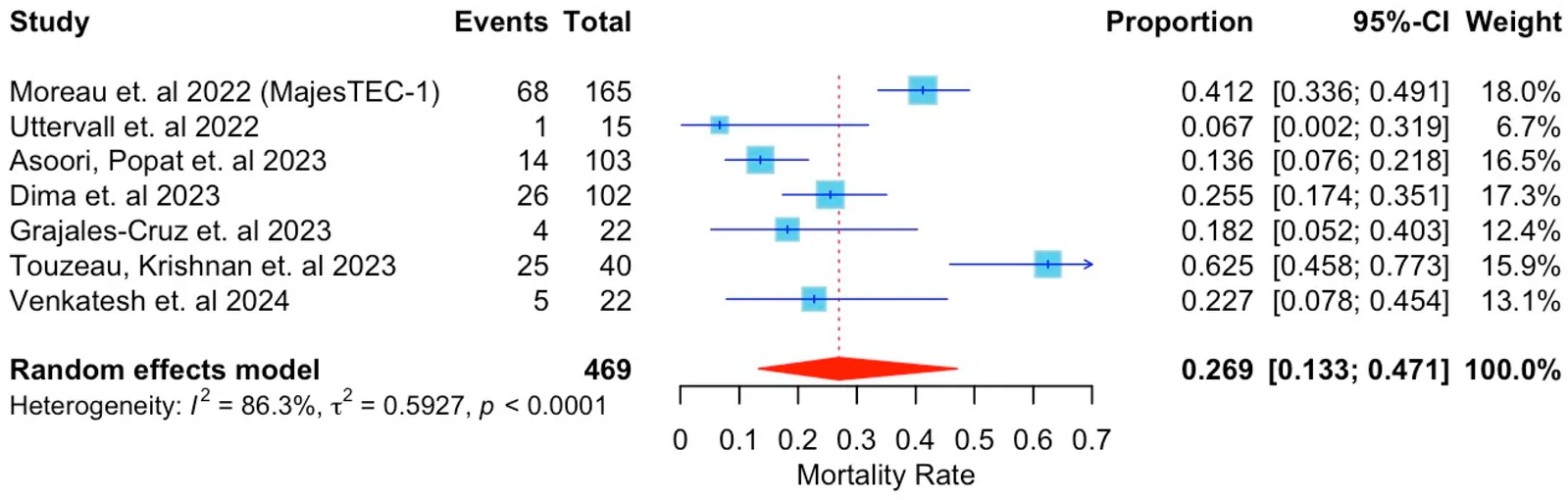
Forest plot of pooled overall mortality rate: 26.9% (95% CI 13.3–47.1%, I^2^ = 86.3%, *p* < 0.0001). Data were derived from contributing studies [9,15,17,18,19,20,25].

**Figure 5:**
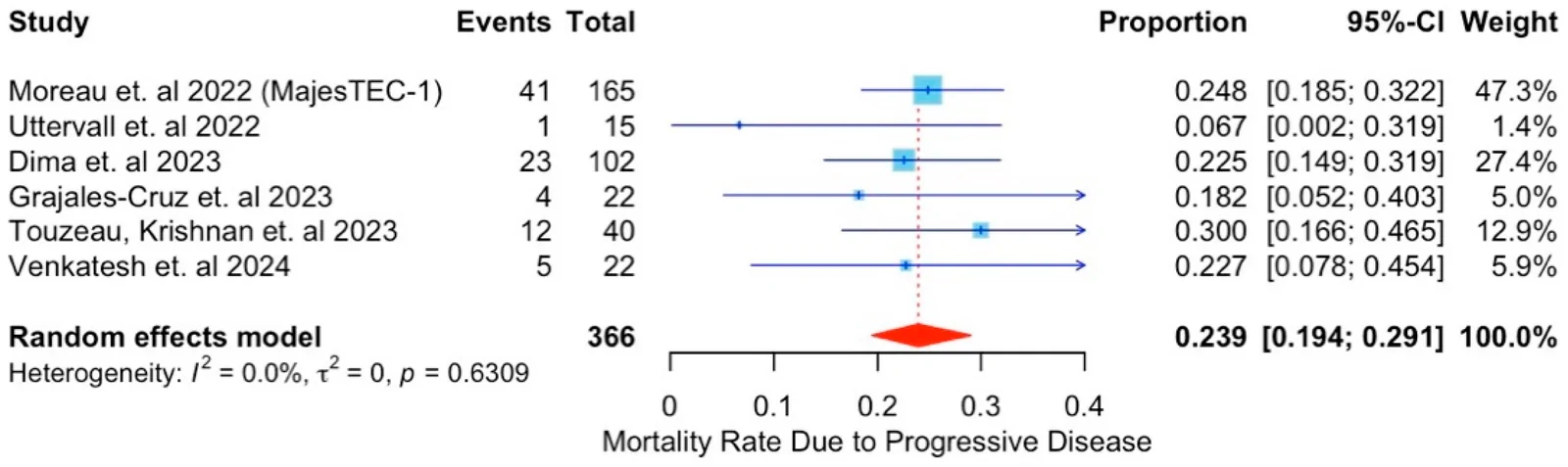
Forest plot of pooled mortality rate due to MM progression: 23.9% (95% CI 19.4–29.1%, I^2^ = 0.0%, *p* = 0.6309). Data were derived from contributing studies [9,15,18,19,20,25].

**Figure 6:**
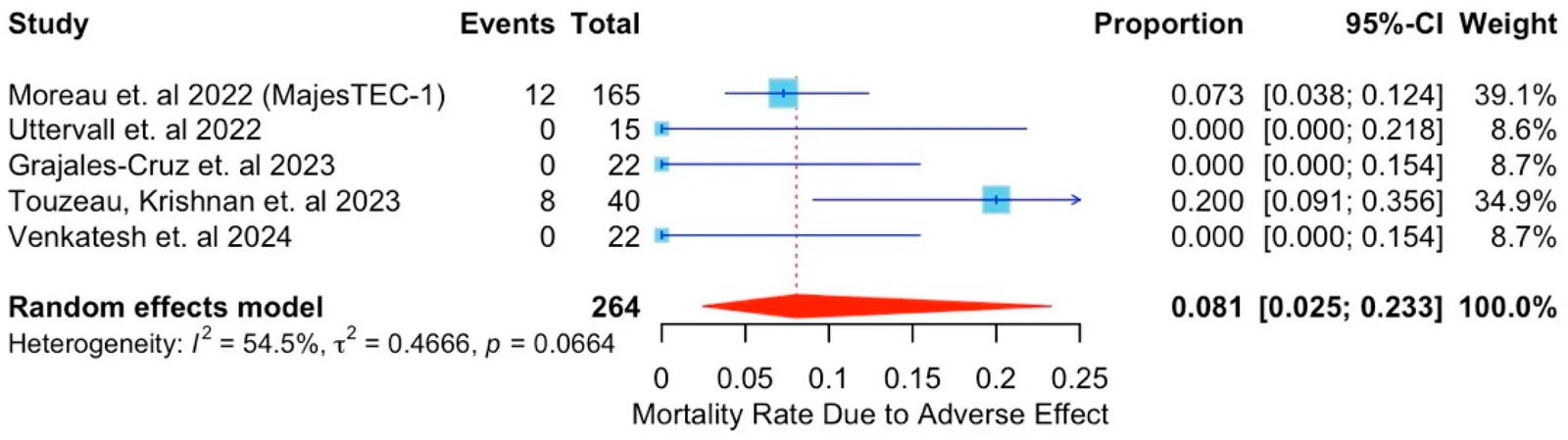
Forest plot of pooled adverse event-related mortality rate: 8.1% (95% CI 2.5–23.3%, I^2^ = 54.5%, *p* = 0.0664). Data were derived from contributing studies [9,15,19,20,25].

**Figure 7:**
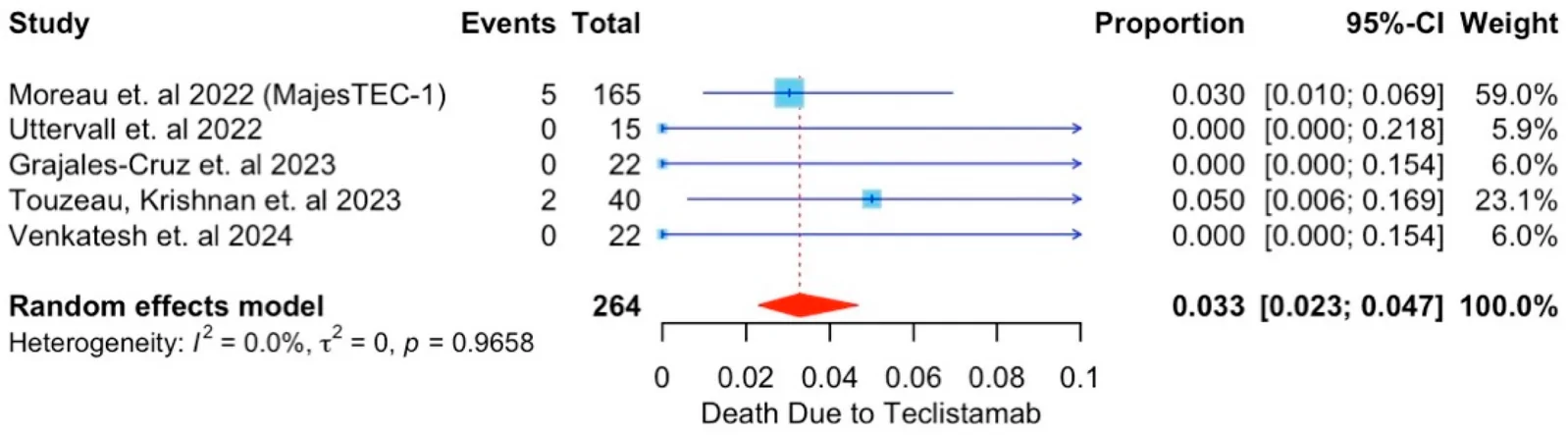
Forest plot of pooled mortality rate directly attributed to teclistamab: 3.3% (95% CI 2.3–4.7%, I^2^ = 0.0%, *p* = 0.9658). Data were derived from contributing studies [9,15,19,20,25].

**Figure 8:**
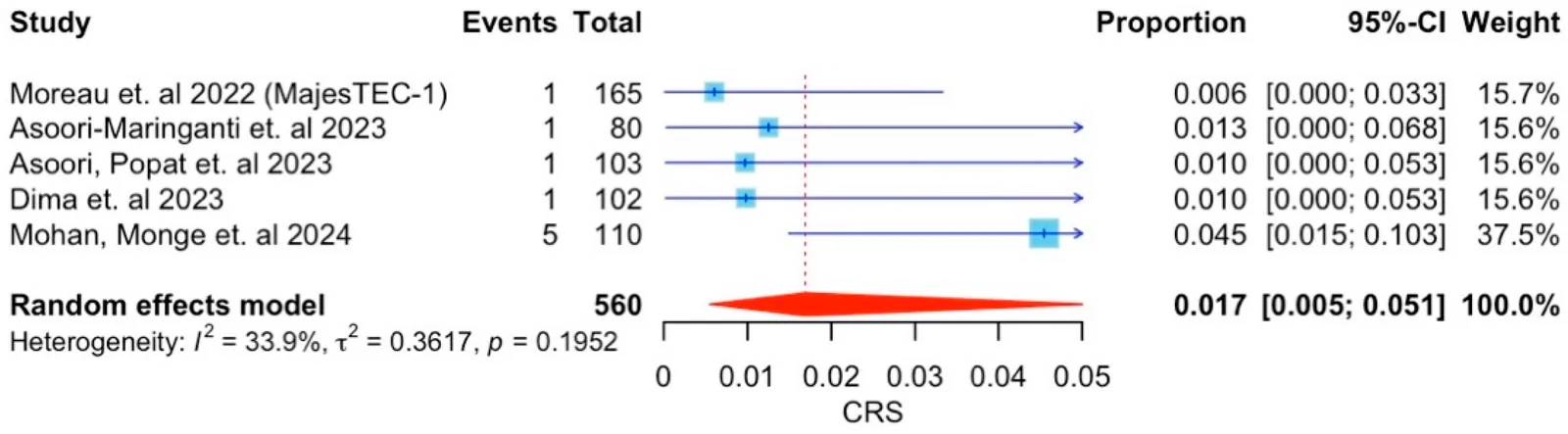
Forest plot of pooled grade ≥3 CRS: 1.7% (95% CI 0.5–5.1%, I^2^ = 33.9%, *p* = 0.1952). Data were derived from contributing studies [9,16,17,18,23].

**Figure 9:**
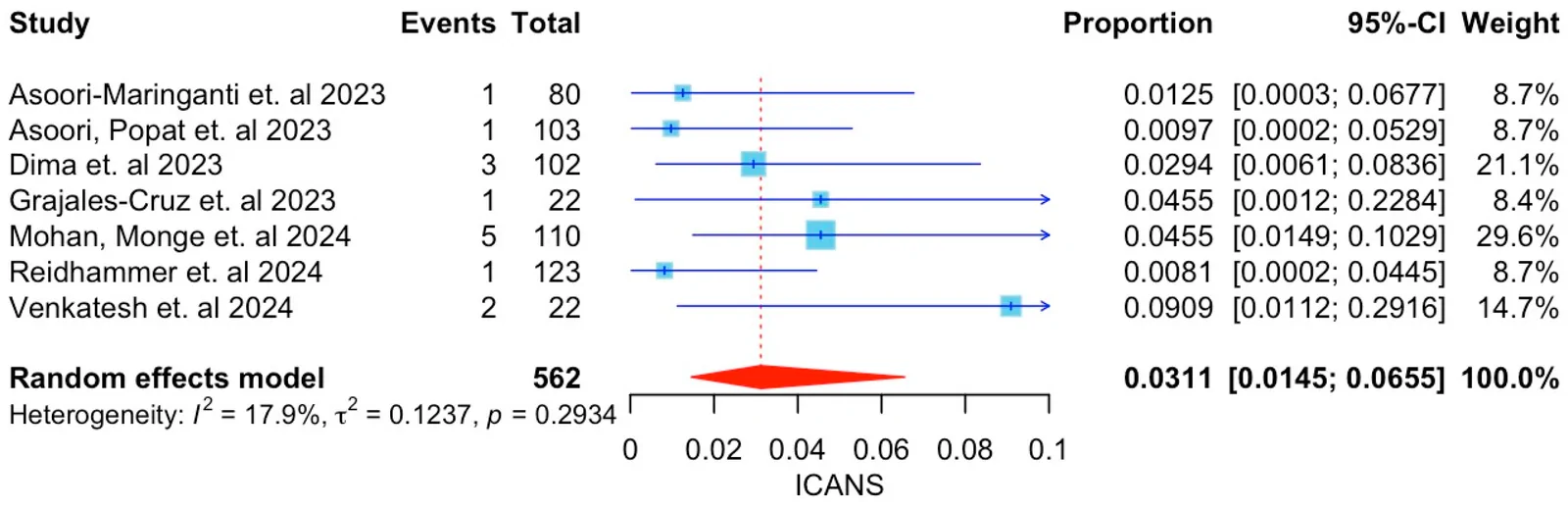
Forest plot of pooled grade ≥3 ICANS: 3.1% (95% CI 1.5–6.6%, I^2^ = 17.9%, *p* = 0.2934). Data were derived from contributing studies [16,17,18,19,23,24,25].

Note for Figure 2, Figure 3, Figure 4, Figure 5, Figure 6, Figure 7, Figure 8 and Figure 9: Blue squares represent individual study estimates, with square size being proportional to study weight. Horizontal lines represent 95% confidence intervals. The red diamond represents the pooled random-effects estimates. The dashed vertical line represents the pooled effect estimate.

### 3.2. Evaluation of Mortality

OMR was analyzed in 7 studies, with a pooled rate of 26.9% (95% CI 13.3–47.1%, I^2^ = 86.3%, *p* < 0.0001) (Figure 4). Mortality due to MM progression was reported in 6 studies. The pooled rate was 23.9% (95% CI 19.4–29.1%, I^2^ = 0.0%, *p* = 0.6309) (Figure 5). Adverse event-related mortality was assessed in 5 studies. The pooled rate was 8.1% (95% CI 2.5–23.3%, I^2^ = 54.5%, *p* = 0.0664) (Figure 6). Mortality directly attributed to teclistamab was reported in 5 studies. The pooled mortality rate was 3.3% (95% CI 2.3–4.7%, I^2^ = 0.0%, *p* = 0.9658) (Figure 7).

### 3.3. Evaluation of Adverse Events

Grade ≥3 CRS and ICANS were analyzed. The pooled incidence of grade ≥3 CRS was 1.7% (95% CI 0.5–5.1%, I^2^ = 33.9%, *p* = 0.1952) (Figure 8), and the pooled incidence of grade ≥3 ICANS was 3.1% (95% CI 1.5–6.6%, I^2^ = 17.9%, *p* = 0.2934) (Figure 9).

Other common grade ≥3 AEs were evaluated. The most common non-hematologic AE was infection, with a pooled incidence rate of 28.3% (95% CI 15.3–46.3%, I^2^ = 78.0%, *p* = 0.0001). Among the most common hematologic AEs, the pooled incidence rates were lymphopenia at 53.0% (95% CI 2.0–99.0%, I^2^ = 85.7%, *p* = 0.0009), neutropenia at 45.0% (95% CI 23.0–69.0%, I^2^ = 92.2%, *p* < 0.0001), anemia at 27.0% (95% CI 15.0–45.0%, I^2^ = 75.6%, *p* = 0.0025), and thrombocytopenia at 23.0% (95% CI 17.0–31.0%, I^2^ = 23.8%, *p* = 0.2626).

### 3.4. Certainty of Evidence

GRADE certainty of evidence was low for ORR, grade ≥3 CRS, and grade ≥3 ICANS. Certainty was very low for CR, OMR, teclistamab-attributed mortality, and grade ≥3 infections, primarily due to variable follow-up duration, outcome-specific ascertainment limitations, imprecision, and suspected publication or reporting bias. The full certainty-of-evidence assessment is provided in Supplementary Table S2.

## 4. Discussion

In this systematic review and meta-analysis of 12 studies including 761 patients, teclistamab monotherapy demonstrated meaningful clinical activity with a manageable safety profile in patients with RRMM. Existing studies have demonstrated teclistamab’s efficacy and safety; however, reported outcomes vary across individual studies due to differences in sample size, study design, and patient populations. This meta-analysis evaluates the consistency of these outcomes across clinical trial and real-world settings.

The pooled efficacy estimates observed in this analysis are broadly consistent with the pivotal MajesTEC-1 trial. In MajesTEC-1, teclistamab demonstrated an ORR of 63.0% and a CR rate of 39.4% at a median follow-up of 14.1 months [9]. In comparison, our pooled ORR of 60.9% remained similar.

A key strength of this analysis is its integration of two prospective clinical trial cohorts with ten retrospective real-world cohorts, providing an updated product-specific summary of teclistamab monotherapy across both controlled and routine practice settings. Prior meta-analyses have summarized teclistamab outcomes in RRMM. Qureshi et al. included five studies and focused on pooled efficacy and selected grade ≥3 safety outcomes, while Li et al. performed a broader analysis that included comparative studies, clinical trials, real-world studies, and teclistamab-containing combination regimens [10,11]. In contrast, the present study focuses specifically on teclistamab monotherapy and provides pooled estimates for efficacy, overall mortality, teclistamab-attributed mortality, immune-mediated toxicity, hematologic toxicity, and infections. This focus is clinically relevant because teclistamab monotherapy remains an important treatment approach in heavily pretreated RRMM, and available real-world evidence continues to expand. These real-world cohorts often include patients with greater comorbidities, higher disease burden, poorer functional status, or other high-risk features that are underrepresented in clinical trials. In addition, this analysis incorporates formal study-level risk-of-bias assessment using a modified Newcastle–Ottawa Scale and certainty-of-evidence assessment using the GRADE framework, providing additional context for interpreting the strength and limitations of the pooled estimates.

Therefore, the consistency of teclistamab’s ORR across broader practice settings suggests that teclistamab retains clinically meaningful activity outside of controlled clinical trial settings. The lower pooled CR rate of 25.2% in our analysis likely reflects the inclusion of broader patient populations with variable disease burden and prior treatments. Substantial heterogeneity was observed for CR, with I^2^ = 71.2%, further reflecting variability in study design, follow-up duration, and response assessment across studies. Overall, these findings suggest that teclistamab efficacy is not limited to clinical trial populations and remains clinically relevant in real-world practice.

While teclistamab demonstrated meaningful efficacy, mortality remained substantial, with a pooled overall mortality rate of 26.9%. Mortality due to myeloma progression was 23.9%, suggesting that disease progression remains a major driver of poor outcomes in this heavily pretreated population. In contrast, mortality directly attributed to teclistamab was relatively low at 3.3%. These findings, however, should be interpreted cautiously, as mortality outcomes were reported inconsistently across studies and were likely influenced by differences in follow-up duration, subsequent therapies, baseline disease risk, and attribution methods. The high heterogeneity observed for overall mortality (I^2^ = 86.3%) reflects this variability across cohorts. Nonetheless, the available data suggest that while teclistamab can produce meaningful responses, patients with RRMM remain at high risk for disease progression and poor outcomes.

An important finding from our safety analysis is the favorable immune toxicity profile of teclistamab. Teclistamab was associated with low rates of severe (grade ≥3) CRS and ICANS in this analysis. The pooled incidence of grade ≥3 CRS was 1.7% and the pooled incidence of grade ≥3 ICANS was 3.1%. These findings suggest that both severe CRS and ICANS are uncommon in patients receiving teclistamab. Although severe CRS and ICANS remain clinically important adverse events requiring early recognition and management, the low pooled rates, along with low pooled teclistamab-attributed mortality, support a manageable immune toxicity profile with appropriate monitoring and established toxicity-management protocols.

While severe CRS and ICANS were rare, severe (grade ≥3) hematologic toxicities and infections were common and represent important clinical considerations. Grade ≥3 lymphopenia, neutropenia, anemia, and thrombocytopenia were the most common hematologic adverse events, with pooled incidence rates of 53.0%, 45.0%, 27.0%, and 23.0%, respectively. Among severe non-hematologic adverse events, grade ≥3 infections were most prominent, with a pooled incidence rate of 28.3%. These toxicities likely reflect several overlapping mechanisms. Heavily pretreated patients with RRMM often have baseline cytopenias due to cumulative effects of prior therapies and disease burden. Teclistamab treatment-related immune or inflammatory effects may then further contribute to these hematologic toxicities [7,8,9]. In turn, these patients are also susceptible to severe infections due to underlying immune dysfunction from myeloma, prior treatment-related immunosuppression, treatment-related cytopenias, and impaired humoral immunity from BCMA-directed plasma cell depletion. These findings also underscore the importance of hematologic monitoring, infection prevention, and early supportive care during teclistamab therapy.

The GRADE assessment further contextualizes these findings. ORR, grade ≥3 CRS, and grade ≥3 ICANS were rated as low certainty, suggesting that the overall pattern of preserved response activity and low rates of severe immune-mediated toxicity was consistent across available studies but remains subject to limitations in study design, reporting, and follow-up. In contrast, CR, grade ≥3 infections, and mortality outcomes were rated as very low certainty, indicating that these estimates should be interpreted with greater caution because they are more susceptible to variation in follow-up duration, response-assessment practices, supportive-care approaches, sparse event counts, and cause-of-death attribution. These findings suggest that although teclistamab monotherapy remains an active and clinically relevant treatment option in RRMM, more standardized prospective data are needed to better define depth and durability of response, infection risk, and longer-term outcomes in real-world populations.

Taken together, this meta-analysis provides a product-specific pooled dataset for teclistamab monotherapy in RRMM. Although this study does not directly compare teclistamab with other bispecific antibodies or CAR-T therapies for RRMM, the observed meaningful response rates and manageable safety profile support teclistamab as a valuable treatment option for patients with RRMM.

## 5. Limitations

This meta-analysis has several important limitations. Because the included studies consisted of two prospective clinical trial cohorts and ten retrospective real-world cohorts, heterogeneity in study design, patient selection, follow-up duration, and outcome reporting was expected. Although median age, sex, prior lines of therapy, and triple-class refractory status were commonly available, cytogenetic risk, extramedullary disease, prior BCMA-directed therapy exposure, disease burden, performance status, and comorbidity data were not consistently reported, precluding reliable subgroup analyses or meta-regression. These analyses should be explored in future studies with larger datasets and more standardized baseline reporting. In addition, safety and mortality outcomes, including infection-related events, hematologic toxicities, follow-up duration, and cause-specific mortality attribution, were not uniformly reported. Because several studies were available only as conference abstracts, methodological detail, follow-up completeness, toxicity attribution, and adjustment for confounding were limited in some cohorts. This systematic review was not prospectively registered in PROSPERO, and a formal protocol was not prepared before study initiation, which may reduce methodological transparency and should be considered when interpreting these findings.

Several additional methodological considerations should be noted when interpreting the pooled estimates. Formal statistical assessment of small-study effects or publication bias using funnel plots or Egger’s test was not performed because many endpoints were based on single-arm pooled proportions with limited numbers of contributing studies, sparse event counts, and substantial clinical heterogeneity, limiting the interpretability of asymmetry-based methods. Sensitivity analyses were not performed because exclusion of abstract-only studies, small cohorts, or potentially overlapping real-world reports would have substantially reduced the number of contributing studies for several outcomes and produced unstable estimates. Certainty-of-evidence assessment using GRADE showed low or very low certainty across key pooled outcomes, primarily due to the predominance of single-arm, non-randomized studies, variable follow-up duration, imprecision, sparse events for several endpoints, limited adjustment for confounding, and inconsistent reporting of several safety and mortality outcomes. Although study-level risk of bias was assessed using a modified Newcastle–Ottawa Scale, causal interpretation remains limited by the single-arm design of the included retrospective studies, which lacked comparator cohorts. In addition, several studies did not include adjusted analyses for age or other clinically relevant prognostic factors, limiting assessment of potential confounding.

Included real-world cohorts were reviewed for potential patient overlap by comparing reported study periods, participating institutions, collaborative groups, and author lists. Although most cohorts appeared institutionally distinct based on available published information, possible overlap among sequential or collaborative abstract reports could not be definitively excluded. Therefore, pooled estimates should be interpreted with recognition that limited duplicate patient contribution across cohorts may have occurred. Furthermore, the present study did not directly compare teclistamab with other bispecific antibodies, CAR-T therapies, or other approved therapies for RRMM, limiting cross-product comparative conclusions. Nonetheless, this product-specific analysis provides valuable pooled estimates that complement prior meta-analyses and reflect the expanding real-world evidence base for teclistamab monotherapy in RRMM.

## 6. Conclusions

This systematic review and meta-analysis provides a product-specific pooled analysis of teclistamab monotherapy in patients with RRMM across clinical trials and real-world settings. Teclistamab demonstrated meaningful pooled response rates, with an ORR of 60.9% and a CR rate of 25.2%, supporting its clinical activity in heavily pretreated patients with RRMM. Severe CRS and ICANS were rare, and mortality directly attributed to teclistamab was low. However, severe hematologic toxicities and infections were frequent and remain important considerations in clinical practice. Overall, these findings support teclistamab as an effective treatment option with a manageable safety profile for patients with RRMM, while underscoring the importance of infection prevention, hematologic monitoring, and supportive care during therapy.

## Figures and Tables

**Table 1:** Baseline characteristics of patients with RRMM treated with teclistamab monotherapy (NR = Not Reported, * reported based on total patients who initiated therapy, total who were evaluable was *n* = 59).

Study (Year)	Evaluable Patients (*n*)	Median Age (Years)	Prior Lines of Therapy, Median	ECOG 0–1, *n* (%)	ECOG ≥2, *n* (%)	Prior BCMA-Directed Therapy Exposure, *n* (%)	Previous Stem Cell Transplant, %	Triple Class Refractory Disease, %
Moreau et al., 2022 [9]	165	64	5	164 (99%)	1 (1%)	0 (0%)	82%	78%
Uttervall et al., 2022 [15]	15	62	9	NR	NR	NR	88%	89%
Asoori-Maringanti et al., 2023 [16]	59	69	6	NR	NR	40/80 (50%) *	NR	77%
Asoori, Popat et al., 2023 [17]	82	63	6	NR	NR	43 (52%)	NR	79%
Dima et al., 2023 [18]	102	66.5	6	73 (72%)	29 (28%)	56 (55%)	NR	92%
Grajales-Cruz et al., 2023 [19]	22	66	8	19 (86%)	3 (13%)	22 (100%)	NR	NR
Touzeau et al., 2023 [20]	40	63.5	6	40 (100%)	0 (0%)	40 (100%)	90%	85%
Firestone et al., 2024 [21]	15	66	7	NR	NR	10 (67%)	NR	100%
Ghamsari et al., 2024 [22]	18	67	6.5	NR	NR	7 (39%)	NR	100%
Mohan et al., 2024 [23]	98	68	6	NR	NR	38 (39%)	87%	86%
Riedhammer et al., 2024 [24]	123	67	6	NR	NR	45 (37%)	NR	93%
Venkatesh et al., 2024 [25]	22	70	6	NR	NR	16 (73%)	NR	91%

## Data Availability

The raw data supporting the conclusions of this article will be made available by the authors on request.

## Supplementary Materials

The following supporting information can be downloaded at: https://www.mdpi.com/article/10.3390/hematolrep18040054/s1, Table S1. Modified Newcastle–Ottawa Scale risk-of-bias assessment for included retrospective observational studies; Table S2. GRADE certainty-of-evidence assessment for key pooled outcomes of teclistamab monotherapy in RRMM. Table S3. PRISMA 2020 Checklist. Reference [12] is cited in Supplementary Materials.
