# Supplementary material for: Teclistamab Monotherapy in Relapsed or Refractory Multiple Myeloma: A Real-World Focused Systematic Review and Meta-Analysis of Efficacy and Safety
Source: Hematol Rep. 2026 Aug 3;18(4):54. doi: 10.3390/hematolrep18040054 (PMC13512276; doi:10.3390/hematolrep18040054)
Supplement: Supplementary file 1 [file hematolrep-18-00054-s001.zip › hematolrep-4347664-Table S1 and Table S2.pdf]

Supplementary Table S1. Modified Newcastle–Ottawa Scale risk-of-bias assessment for included retrospective observational studies.

Author, Year	Study Design	Representativeness	Non-exposed cohort	Exposure ascertainment	Outcome absent at baseline	Compa rability	Outcome assessment	Follow-up duration	Follow-up adequacy	Total	Risk of bias
Uttervall et al., 2022	Retrospective, multi-center	★	—	★	★	—	★	★	★	6	Moderate
Maringanti et al., 2023	Retrospective, multi-center	★	—	★	★	★	★	★	—	6	Moderate
Asoori, Popat et al., 2023	Retrospective, multi-center	★	—	★	★	★	★	★	—	6	Moderate
Dima et al., 2023	Retrospective, registry-based	★	—	★	★	★★	★	★	★	8	Low
Grajales-Cruz et al., 2023	Retrospective, single-center	★	—	★	★	—	★	★	★	6	Moderate
Firestone et al., 2024	Retrospective, single-center	★	—	★	★	★	★	★	—	6	Moderate
Ghamsari et al., 2024	Retrospective, single-center	★	—	★	★	★	★	★	★	7	Low
Mohan et al., 2024	Retrospective, multi-center	★	—	★	★	★★	★	★	★	8	Low
Riedhammer et al., 2024	Retrospective, multi-center	★	—	★	★	★★	★	★	★	8	Low
Venkatesh et al., 2024	Retrospective, single-center	★	—	★	★	★	★	★	★	7	Low

Note: Modified Newcastle–Ottawa Scale for single-arm observational teclistamab studies. ★ = criterion met; — = not applicable/no comparator cohort. Comparability was scored only when age and/or other prognostic factors were adjusted for or analyzed. Low risk = 7–9; moderate = 4–6; high = 0–3.

Supplementary Table S2. GRADE certainty-of-evidence assessment for key pooled outcomes of teclistamab monotherapy in RRMM.

Outcome	Studies/ Patients (*n*)	Pooled estimate, % (95% CI)	Starting certainty	Risk of bias	Inconsisten cy	Indirectness	Imprecision	Publication bias	Final certainty
ORR	12/761	60.9 (58.5–63.3)	Low	Not serious	Not serious	Not serious	Not serious	Not serious	⊕⊕◯◯ Low
CR	9/713	25.2 (18.8–32.9)	Low	Serious[a]	Serious[b]	Not serious	Serious[c]	Suspected[d]	⊕◯◯◯ Very Low
OMR	7/469	26.9 (13.3–47.1)	Low	Serious[e]	Serious[f]	Not serious	Serious[c]	Suspected[d]	⊕◯◯◯ Very Low
Mortality attributed to teclistamab	5/264	3.3 (2.3–4.7)	Low	Serious[g]	Not serious	Serious[h]	Serious[i]	Serious[d]	⊕◯◯◯ Very Low
Grade ≥3 CRS	5/560	1.7 (0.5–5.1)	Low	Not serious	Not serious	Not serious	Not serious	Not serious	⊕⊕◯◯ Low
Grade ≥3 ICANS	7/562	3.1 (1.5–6.6)	Low	Not serious	Not serious	Not serious	Not serious	Not serious	⊕⊕◯◯ Low
Grade ≥3 Infections	7/440	28.3 (15.3–46.3)	Low	Serious[j]	Serious[k]	Not serious	Serious[c]	Not serious	⊕◯◯◯ Very Low

Notes: Certainty was assessed using the GRADE framework. Because evidence base consisted predominantly of single-arm, non-randomized studies, all outcomes started at low certainty. Certainty reflects confidence in pooled absolute estimates rather than comparative treatment effects.

[a] Downgraded because CR assessment may be affected by variable follow-up and incomplete confirmatory assessment in real-world studies.
[b] Downgraded for heterogeneity in depth-of-response reporting.
[c] Downgraded for wide confidence intervals.
[d] Downgraded or suspected because several outcomes were reported by small or abstract-only studies.
[e] Downgraded because mortality estimates were affected by variable follow-up and retrospective ascertainment.
[f] Downgraded for heterogeneity in baseline disease risk and follow-up duration.
[g] Downgraded because attribution of death to teclistamab was difficult and inconsistently reported.
[h] Downgraded because studies did not uniformly define or adjudicate teclistamab-attributed mortality.
[i] Downgraded because the estimate was based on few events.
[j] Downgraded because infection ascertainment and grading varied across studies.
[k] Downgraded for substantial heterogeneity in infection reporting.
